# A Longitudinal 3D Investigation on Facial Similarity among Two Monozygotic Twins in Their First Childhood: An Application of the 3D-3D Facial Superimposition Technique

**DOI:** 10.3390/children9020187

**Published:** 2022-02-02

**Authors:** Daniele M. Gibelli, Annalisa Cappella, Claudia Dolci, Riccardo Rosati, Marzia Bedoni, Chiarella Sforza

**Affiliations:** 1 Department of Biomedical Sciences for Health, Università degli Studi di Milano, 20133 Milano, Italy; daniele.gibelli@unimi.it (D.M.G.); annalisa.cappella@unimi.it (A.C.); claudia.dolci@unimi.it (C.D.); riccardorosati@outlook.com (R.R.); 2 U.O. Laboratorio di Morfologia Umana Applicata, IRCCS Policlinico San Donato, 20097 San Donato Milanese, Italy; 3 IRCCS Fondazione Don Carlo Gnocchi Onlus, 20148 Milano, Italy; mbedoni@dongnocchi.it

**Keywords:** facial anatomy, monozygotic twins, children, stereophotogrammetry

## Abstract

Children affected by orofacial disorders mix functional alterations with morphological problems, and suitable techniques should be devised for their analysis. Stereophotogrammetry and 3D-3D facial superimposition have already proven to reliably assess morphological differences even between twin siblings, separating the effect of genetic and environmental factors. However, little information is available about twin babies. We longitudinally analyzed a couple of healthy monozygotic twin sisters aged 6 months to 5 years (height time points). The entire 3D facial models of the two sisters were registered according to the least point-to-point distance, and the relevant RMS (root mean square) distance between the facial models was calculated at each time and compared with reference data recorded from adult twins (Mann-Whitney test, *p* < 0.05). RMS values in the twin sisters were on average 1.18 ± 0.21 mm, and 1.86 ± 0.53 mm in adults, with a significant difference (*p* < 0.01). Results showed that twins are more similar in early childhood when environmental factors are supposed to have not influenced facial morphology sufficiently. Additionally, the technique seems adequate to detect even small differences: the faces of the twin sisters were not fully identical. 3D-3D facial superimposition techniques can objectively quantify facial dissimilarity even in monozygotic twins. The method may be applied to the faces of twins discordant for some orofacial and maxillofacial pathology and potentially separate genetic and environmental factors.

## 1. Introduction

Morphological analysis of face has importance in several fields of research, including the diagnosis of genetic and congenital diseases, assessment of results after aesthetic and maxillofacial surgery, and evaluation of facial modifications due to orthognathic procedures [1,2,3,4]. With time, facial analysis was strongly improved by the introduction of optical 3D surface acquisition systems and the relevant measurement tools [5]: one of them is represented by stereophotogrammetry, whose accuracy and repeatability have been already positively assessed by literature [6,7]. In detail, 3D acquisition techniques allow operators to obtain 3D facial models which can be superimposed one on each other: in this way, differences between facial surfaces can be expressed as RMS (root mean square) point-to-point distance, which may be used as a parameter for assessing similarities between different faces or the same across time during normal growth or treatment follow-up [8,9]. Stereophotogrammetry does not use ionizing radiations or dangerous and painful procedures, an essential aspect when longitudinal examinations are planned.

A challenging field of application of facial analysis concerns the examination of monozygotic twins: in fact, they share the same genetic alleles, but can be influenced by different environmental determinants (i.e., discordance for some malformation in the dental and maxillofacial areas) which might produce instead dissimilarities at all levels, including facial morphology [3,10,11]. Environmental variability may involve hormonal adaptive response, nutrition, congenital and acquired diseases, abnormalities and malformations, traumatic and surgical lesions, and lifestyle variables [4]. Gene expression and molecular interactions act initially in directing the complicated developmental process of facial structures at the embryonic level, representing during this period the main actors. On the contrary, hormones and environmental determinants play an important role in the facial growth and its final appearance mainly during the childhood and puberal periods [12,13,14]. 

In these terms, the influence’s strength, determined by genetic and environmental factors, in the final facial morphology remains important to elucidate. Its debate has already posed the light on the need to properly research what the relative contributions given by genetic and environmental components to the facial morphology are, and recently has started to receive much attention.

The dynamic and complicated phenomena of development and growth of the facial soft tissues have conventionally been studied on photographs and anteroposterior or lateral cephalograms, and only recently analyzed on 3D facial models acquired through technological systems such as laser scanner and stereophotogrammetry [15,16].

In these terms, 3D facial analysis of monozygotic twins may therefore provide interesting information about the interaction between genes and environment [17,18] and much more data in comparison with those deriving from 2D facial analysis. For instance, through the acquisition of 3D facial models by laser scanner and/or stereophotogrammetry, the 3D analysis might exploit the comparison/superimposition of facial surfaces of twins, providing values of average distances existing between the two superimposed models. This approach was already implemented in a recent descriptive observational study conducted in adult twins, which reported reference data about facial similarity [18] without, however, any interpretation on the potential contribution that environmental factors might play in producing differences. Indeed, this type of 3D-analysis offers to all effects a practical and objective investigation for quantifying facial dissimilarity existing between monozygotic twins discordant for some orofacial and maxillofacial pathology and potentially due to environmental factors, thus providing important clues on the relative environmental contribution to the definitive facial morphology.

Over the last years, the 3D investigation on facial similarity in twins has gained increasing attention; however, studies focusing exactly on facial morphology as a key to understanding the effects of environmental factors are still few in adults and adolescent twins, and absent for the early childhood (between 0 and 6 years). Specifically, some 3D cross-sectional studies in adult and adolescent twins have been conducted with the intent to underline differences in facial similarity between monozygotic and dizygotic twins [19,20,21]. However, except for Djordjevic et al. [17], who also performed a surface-based superimposition analysis, all these studies took into consideration anthropometric distances, facial shape, and/or symmetry of facial features, aiming particularly to understanding the contribution played by genetic (zygosis) in facial similarity rather than by environmental determinants.

In fact, to possibly generate hypotheses about the effect that environmental determinants have on the final facial phenotype, the investigation should mainly focus on the longitudinal facial analysis of pairs of monozygotic twins (starting from birth). In such research, one could monitor the similarity during growth, focusing uniquely on the role that pathology and/or environmental factors play in facial growth and directing the facial morphological variation among the two twins.

To the best of our knowledge, only one study [22] reported in the literature provided data on the facial morphology of monozygotic children twins longitudinally examined (over a period ranging between 9 and 18 years old); although the authors examined several pairs of growing twins, their aim was to verify the variation on facial symmetry over time, and no evidence on potential changes of facial similarity during growth was provided at that time. So, no data have been yet reported on facial similarity for monozygotic twin children, and on how such a similarity may vary over time during growth, especially through a quantitative and objective 3D analysis. Through this study, we therefore wish to provide preliminary data regarding the variation of similarity of facial morphology in a pair of healthy monozygotic twins over the first early childhood, with the intent to quantify differences, if any, and how and when they change during the first years of life.

In particular, this study provides results from a 3D-3D superimposition approach from 3D facial models of a couple of monozygotic twin sisters longitudinally acquired through stereophotogrammetry between 6 months and 5 years old. The obtained data were compared with reference values of facial similarity existing among monozygotic adult twins [18] in order to assess possible variations in facial morphology between monozygotic twins at different ages.

## 2. Materials and Methods

A couple of monozygotic twin sisters, at the age of six months, was recruited for the study. The two sisters were not affected by genetic, congenital, or acquired pathologies, nor by possible traumatic lesions or surgical procedures. They were born by caesarian delivery at the 33rd week of a monochorionic diamniotic pregnancy; feeding and weaning were identical.

The experimental protocol for 3D facial acquisition was not invasive or dangerous. The experimental study followed the Helsinki Declaration (seventh edition, 2013). The study was also approved by the University of Milan ethics committee (Comitato Etico Unimi 26 March 2014—No. 92/14).

**Figure 1:**
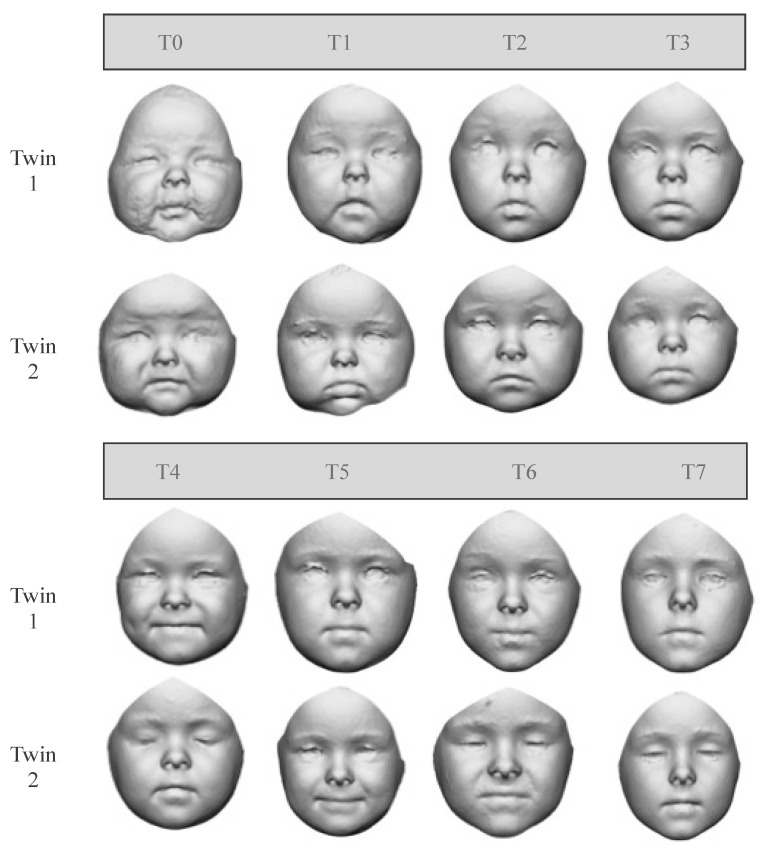
Details of each FAI from the twin sisters at each time of acquisition: T0 = 6 months, T1 = 9 months, T2 = 13 months, T3 = 17 months, T4 = 26 months, T5 = 36 months, T6 = 50 months, and T7 = 63 months. Twin 1: first born; Twin 2: second born.

The two twin sisters were observed through a stereophotogrammetric system (VECTRA-3D^®^ M3: Canfield Scientific, Inc., Fairfield, NJ, USA), an optical instrument with a geometry resolution of 1.2 mm [23] at different time points after birth: six (T0), nine (T1), 13 (T2), 17 (T3), 26 (T4), 36 (T5), 50 (T6), and 63 (T7) months (Figure 1). Facial scans were acquired on the participants sitting on their relatives’ laps, with repeated acquisitions to obtain the optimal one: with a rest position, closed mouth, and neutral facial expression [24].

From each facial model, a FAI (facial area of interest) was selected using the following ten anatomical landmarks: trichion, gnathion and right and left frontotemporale, zygion, tragion, and gonion. The procedure of FAI selection has already been applied in previous studies and was found to be well repeatable [25]. The obtained FAIs were further analyzed through VAM^®^ software (Canfield Scientific, Inc., Fairfield, NJ, USA): in detail, at each time period, FAIs belonging to the two sisters were registered and superimposed one on each other according to the least point-to-point distance between the two models. Conventionally, the face of the second-born twin was superimposed on the face of the first.

**Figure 2:**
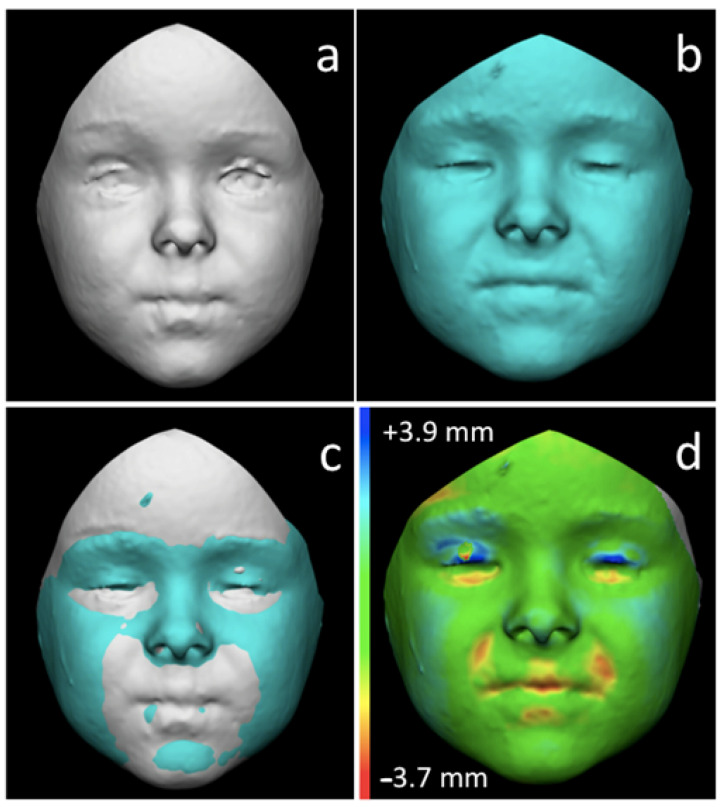
Steps of 3D-3D registration of two FAIs belonging to the twin sisters at T6: (**a**) FAI of the first-born twin; (**b**) FAI from the second-born twin; (**c**) registration of the two surfaces according to the least point-to-point distance between the two FAIs; (**d**) calculation of RMS (root mean square) point-to-point distance between the two models, using the first born twin as reference: blue areas are protruding in the second-born twin according to the first-born one; red areas are recessing in the second-born twin according to the first-born one; green areas are concordant in both the twins.

After the superimpositions were performed, the RMS (root mean square) point-to-point distance was automatically calculated between the corresponding points of the two facial surfaces on the entire FAI. The first-born twin was chosen as reference for the calculation of RMS (Figure 2). 

Values of RMS distances acquired from the twin sisters at the different time periods were then compared with reference values of facial similarity of monozygotic adult twins. Such values were obtained through the same 3D experimental protocol and were reported in a previous publication [18]. The comparison between RMS values of the two monozygotic twin sisters under study and adult twins was performed by the calculation of z-scores according to the following formula:

z-score = (x − µ) σ(1)

where x is the value of each measurement calculated in the twin sisters, and µ and σ are respectively the mean and standard deviation of the same measurement computed on the adult twins. The smaller the z-score, the closer the twin sisters’ RMS values to the adult ones. Possible statistically significant differences in z-score for each measurement between twin children and adults were assessed through Mann-Whitney test (*p* < 0.05).

## 3. Results

Descriptive statistics of RMS values at the different time periods are shown in Table 1, with corresponding z-scores obtained by the comparison with data from adult twins. RMS values for the twin sisters ranged from −6.76 mm (T5) to 6.16 mm (T1), with mean values up to 0.32 mm (T3). In the twin sisters, RMS distances were on average 1.18 ± 0.21 mm, while 1.86 ± 0.53 mm was the average RMS value found in adult twins [18]. Z-scores of RMS distances in twin sisters ranged between 0.9 and 1.9, with a significant difference in comparison with the adult population (*p* < 0.01).

## 4. Discussion

Morphological analysis of the face, which recently reached great improvements thanks to the advent of 3D new technologies, represents an important field of research, applicable to a plethora of contexts including clinical, diagnostic, forensic, and surgical ones [1,2,3,4,5,6,7]. Moreover, 3D acquisition techniques allow for drawing objective and quantified parameters of dissimilarities and similarities existing among two faces. 

The concepts of similarity and dissimilarity can be very useful for the analysis of facial modifications, for instance, to detect changes due to malformations or produced by a surgical procedure; to track facial changes related to growth; to correlate facial features to peculiar genetic and congenital diseases; and furthermore also in the field of personal identification as, for example, for identifying kidnapped children released after years or main suspects recorded from video surveillance systems, just to mention some [26]. 

In the assessment of monozygotic twins, 3D-3D registration and superimposition procedures may help in analyzing differences among and within subjects, allowing quantification, or at least to approximation, of the impact of environmental factors in the construction of the “definitive” adult facial morphology, as the genetic component is minimized in twins who share 100% identical genetic materials. From this point of view, the 3D approach that calculates the RMS point-to-point distance may represent a valid technique for quantifying differences between subjects sharing the same genetic material, as also confirmed by the literature [24].

The 3D acquisition systems have already been applied to adult twins through both laser scanners and stereophotogrammetry [17,18]. In detail, the stereophotogrammetric study was performed by our research group: results showed that facial differences in cases of monozygotic twins were higher in terms of RMS values in comparison with superimpositions involving 3D models from the same individual, but lower than those obtained by the superimposition of 3D facial models of unrelated subjects. Similar results were confirmed by Djordjevic et al. through laser scanner acquisitions, who found statistically significant differences between the average RMS value of monozygotic and dizygotic twins: the RMS value was inferior in monozygotic twins (0.82 mm) relative to dizygotic twins (1.30 mm) during their adolescence [10,17]. Unfortunately, these results are not comparable with the previous study as the registration was performed between scaled models according to Procrustes procedures [10]. However, so far no study has been published about the 3D comparison of faces of twins in their early childhood (first years of life after birth). 

The present study is the first to present data on the longitudinal facial comparison of a couple of twin sisters followed from six months after birth up to five years old. Results showed that facial differences between the twin children, expressed as RMS values, were significantly lower than the similar data recorded in adult twins, i.e., monozygotic twins seem to show more facial similarity in their childhood than in adolescence and adulthood, resembling other more when babies rather than adults. This result may be justified by the reduced influence of environmental factors in infant rather than in adult ages, resulting in a more pronounced similarity between the two sisters [20,22,27,28]. Indeed, the current RMS values were lower than the average RMS values recorded in adults by up to 1–2 standard deviations (z-score values), but the result was obtained in a single pair of twins and, though appealing, it should be verified in a larger sample.

The proposed technique may find another important application in the forensic field, as 3D-3D superimposition techniques will be found in the future as an additional use in case of identification of subjects represented by video surveillance systems: a previous study by Gibelli et al. [26] found that 3D models belonging to the same individual usually show an RMS value lower than 1.50 mm. Interestingly, in all the eight acquisitions, the two sisters showed an RMS value lower than the reported threshold. Nonetheless, the faces of the twin sisters were not fully identical, and the RMS values obtained by the 3D-3D superimposition showed that each of them possessed some specific characteristics that can be used for their differentiation.

The present study has some limits which need to be adequately discussed: first, results were extracted from a single couple of healthy twins. From this point of view, further analyses on wider samples are crucial, although a longitudinal study on twins since the post-natal period may be difficult to manage. Secondarily, for the first time, 3D acquisition systems were used on six-month-old subjects, with obvious limitations concerning involuntary movement and mimicry [29]. From this point of view, stereophotogrammetry is undoubtedly superior to the laser scanner, as the former method provides a model of the entire face in only one acquisition (3.5 ms), whereas the latter method may require multiple acquisitions or need a longer scanning time [5]. However, involuntary facial mimicry is difficult to limit in case of 3D acquisition of the youngest children: in the present study, multiple acquisitions were performed in order to obtain the ideal facial asset, namely as close as possible to the rest position (close mouth and neutral facial expression), usually requested in acquisitions of adult subjects. Thus, although the use of 3D acquisition systems in youngest children proves to be very useful, the provided data should still be considered with caution. Additionally, the analysis was performed on all facial surfaces, while other investigations divided it in into thirds [17,25]: the reduced dimensions of baby faces prevented further subdivisions that may be performed in older children. Furthermore, it should be noted that the average RMS distance obtained by the eight comparisons (1.18 mm) was similar to the resolution of the acquisition technology (1.2 mm). It means that the “real “average RMS distance could potentially be between 0 and 2.38 mm.

## 5. Conclusions

In conclusion, the 3D-3D stereophotogrammetry technique described in the present study provides a valid, efficient, and objective method to quantify morphological facial similarity among monozygotic twins, allowing to appreciate the magnitude of similarities and differences between two superimposed faces. The assessment of similarity of facial morphology in a couple of monozygotic twin sisters, followed-up from the age of six months after birth to five years, provided support to the hypothesis that monozygotic twins are more similar one to each other in childhood rather than in adult age, suggesting that heritability has a strong contribution still persisting in the early childhood. Future studies focused on facial similarity in monozygotic twins longitudinally followed from their middle childhood to early adolescence could shed light on the relationship between genetic and environmental factors in determining the “definitive” facial morphology, helping us to better understanding this complex mechanism.

Additionally, the technique seems adequate to detect even small differences among and within patients using safe and comfortable procedures that can be repeated over time: the faces of the twin sisters were not fully identical. Similarities and differences were quantified by the RMS values obtained by the 3D-3D superimposition technique, demonstrating that each sister possessed some specific characteristics. The 3D-3D superimposition can objectively quantify facial dissimilarity even in monozygotic twins. The method may be applied to the faces of twins discordant for some orofacial and maxillofacial pathology and potentially separate genetic from environmental factors.

## Figures and Tables

**Table 1:** RMS, mean, minimum (min), maximum (max), and standard deviation of point-to-point distance between the two models in eight different time points. The corresponding z-scores obtained by the comparison with data from adult twins are reported.

Time	RMS	Max	Min	Mean	SD	z-Score
T0	1.36	3.43	−3.99	0.29	1.33	0.9
T1	1.12	6.16	−5.19	0.08	1.12	1.4
T2	1.33	4.86	−4.20	0.29	1.30	1.0
T3	1.08	3.42	−2.99	0.32	1.03	1.5
T4	1.35	6.11	−5.29	−0.15	1.34	1.0
T5	1.40	3.89	−6.76	0.27	1.38	0.9
T6	0.95	3.88	−3.74	0.18	0.94	1.7
T7	0.85	2.66	−3.58	0.17	0.83	1.9

## Data Availability

The data that support the findings of this study are available on request from the corresponding author. The data are not publicly available due to privacy and ethical restrictions.
